# Evaluation of the effect of experimentally induced cartilage defect and intra-articular hyaluronan on synovial fluid biomarkers in intercarpal joints of horses

**DOI:** 10.1186/s13028-019-0460-6

**Published:** 2019-05-30

**Authors:** Tytti Maaria Niemelä, Riitta-Mari Tulamo, Jorge Uriel Carmona, Catalina López

**Affiliations:** 1 Department of Equine and Small Animal Medicine, Faculty of Veterinary Medicine, University of Helsinki, P.O. Box 57, 00014 Helsinki, Finland; 2 Grupo de Investigación Terapia Regenerativa, Departamento de Salud Animal, Universidad de Caldas, Calle 65 No26-10, Manizales, Caldas, Colombia

**Keywords:** Experimental study, Cartilage defect, Hyaluronan, Interleukin-1 receptor antagonist, Non-animal stabilized hyaluronic acid (NASHA), Platelet-derived growth factor BB, Synovitis, Transforming growth factor β_1_, Tumour necrosis factor α

## Abstract

**Background:**

Inflammatory and degenerative activity inside the joint can be studied in vivo by analysis of synovial fluid biomarkers. In addition to pro-inflammatory mediators, several anabolic and anti-inflammatory substances are produced during the disease process. They counteract the catabolic effects of the pro-inflammatory cytokines and thus diminish the cartilage damage. The response of synovial fluid biomarkers after intra-articular hyaluronan injection, alone or in combination with other substances, has been examined only in a few equine studies. The effects of hyaluronan on some pro-inflammatory mediators, such as prostaglandin E_2_, have been documented but especially the effects on synovial fluid anti-inflammatory mediators are less studied. In animal models hyaluronan has been demonstrated to reduce pain via protecting nociceptive nerve endings and by blocking pain receptor channels. However, the results obtained for pain-relief of human osteoarthritis are contradictory. The aim of the study was to measure the synovial fluid IL-1ra, PDGF-BB, TGF-β_1_ and TNF-α concentrations before and after surgically induced cartilage defect, and following intra-articular hyaluronan injection in horses. Eight Standardbred horses underwent bilateral arthroscopic surgeries of their intercarpal joints under general anaesthesia, and cartilage defect was created on the dorsal edge of the third carpal bone of one randomly selected intercarpal joint of each horse. Five days post-surgery, one randomly selected intercarpal joint was injected intra-articular with 3 mL HA (20 mg/mL).

**Results:**

Operation type had no significant effect on the synovial fluid IL-1ra, PDGF-BB, TGF-β_1_ and TNF-α concentrations but compared with baseline, synovial fluid IL-1ra and TNF-α concentrations increased. Intra-articular hyaluronan had no significant effect on the biomarker concentrations but a trend of mild improvement in the clinical signs of intra-articular inflammation was seen.

**Conclusions:**

Creation of the cartilage defect and sham-operation lead to an increase of synovial fluid IL-1ra and TNF-α concentrations but changes in concentrations of anabolic growth factors TGF-β_1_ and PDGF-BB could not be documented 5 days after the arthroscopy. Intra-articular hyaluronan was well tolerated. Further research is needed to document possible treatment effects of intra-articular hyaluronan on the synovial fluid biomarkers of inflammation and cartilage metabolism.

## Background

Research on equine joint disease has been focused on finding tools for early diagnosis and monitoring the treatments and progression of the joint disease. Inflammatory and degenerative activity in the joint can be studied in vivo by analysis of synovial fluid (SF) biomarkers, such as pro-inflammatory cytokines in experimental setting [1–5], or in naturally occurring joint disease [6–8]. In addition to pro-inflammatory mediators, several anabolic and anti-inflammatory mediators are produced during the inflammatory process. They counteract the catabolic effects of the pro-inflammatory cytokines and diminish the cartilage damage. Moreover, the anti-inflammatory effect can produce temporal relief of the clinical symptoms of the articular disease [9].

Equine arthritis is commonly treated with intra-articular (IA) corticosteroids and hyaluronan (HA) [10]. However, the response of SF biomarkers after IA HA injection, alone or in combination with other substances, has been examined only in a few equine studies [3, 4, 11]. In human medicine, clinical efficacy of HA has been widely studied [12]. In addition, the effect of IA HA on some SF pro-inflammatory biomarkers, such as prostaglandin E_2_, has been explored both in human and equine studies [3, 4, 13–15]. However, research on the effect of HA on anti-inflammatory mediators has been infrequently conducted. Despite the observed effects of HA on pro-inflammatory mediators, results in pain-relief in human osteoarthritis (OA) are contradictory [12]. In animal models HA has been demonstrated to reduce pain via protecting nociceptive nerve endings [16] and by blocking pain receptor channels [17].

The aim of the study was to measure SF concentrations of the anti-inflammatory mediator interleukin-1 receptor antagonist (IL-1ra), the anabolic growth factors platelet-derived growth factor BB (PDGF-BB) and transforming growth factor beta-1 (TGF-β_1_) and the pro-inflammatory cytokine tumour necrosis factor alpha (TNF-α) before and after surgically induced cartilage defect (CD) in healthy horses. Secondly, we wanted to explore if the concentrations of the selected biomarkers changed following the IA HA (non-animal stabilized hyaluronic acid, NASHA^1^) injection. The hypothesis was that both concentrations of anti-inflammatory cytokines and concentration of TNF-α will increase in SF after the induction of CD; and that in the HA-injected joints the pro-inflammatory TNF-α is decreased and the concentrations of anti-inflammatory mediators are increased compared with the joints without the HA medication.

## Methods

The study protocol was approved by the National Animal Experimental Board in Finland. Eight Standardbred horses (four mares, one stallion and three geldings) free of lameness were used. Before recruiting the horses, combination of interventions was randomly picked for each right intercarpal joint. As a result, each joint was selected for one of the following; cartilage defect (CD) with HA, CD without injection, sham-operation (SO) with HA or SO without injection. Interventions for each of the contralateral intercarpal joints were determined by these randomly picked combinations; i.e. if CD with HA was selected for the right side, left side was for SO without injection.

The median age of horses was 7 years and range 4–24 years. Examinations, grading of measured variables and surgical procedures were carried by the principal veterinarian (TMN). Prior to inclusion, horses were subjected to a complete lameness examination. A standardized American Association of Equine Practitioners’ (AAEP) scale of 0–5 [18] was used to grade lameness. Effusion of the affected joint was recorded on a scale of 0–4 (0 = no effusion, 1 = mild, 2 = moderate, 3 = severe effusion, 4 = severe swelling of the joint region) [19]. A flexion test of the affected and the contralateral limb was performed, and lameness was recorded on a scale of 0–4 (0 = no increase, 1 = slight increase, 2 = moderate increase, 3 = considerable increase compared with the baseline lameness, 4 = non-weight-bearing lameness) [3]. Pain score for maximal flexion of the carpus was also determined and recorded on a scale of 0–3 (0 = no pain on flexion, 1 = mild pain, i.e. the horse shows some reaction, such as moving the limb, 2 = moderate pain, i.e. the horse retracts the limb repeatedly during the 1 min flexion period, 3 = severe pain, i.e. the flexion test cannot be properly performed). In addition, five radiographic views (dorsopalmar, dorsolateral-palmaromedial, dorsomedial-palmarolateral, flexed lateromedial and flexed dorsoproximal-dorsodistal) of the carpal joints were assessed.

Before the surgical procedure, 5 mL of the SF of both intercarpal joints of each horse were aspirated into a sterile 5 mL syringe for the biomarker measurements. The SF sample was immediately divided between a plain 4 mL tube on ice and into an etylenediaminetetraacetic acid (EDTA) tube. White blood cell (WBC) count and total protein (TP) concentration measurements were done from the fresh sample in the EDTA tube. Within 1 h of collection, the plain sample was centrifuged at 4000 rpm for 10 min in 4 °C, aliquoted and stored at − 80 °C.

The horses underwent bilateral arthroscopic surgeries of their intercarpal joints under general anaesthesia. No pre-existing IA abnormalities were detected during the arthroscopy in any of the joints. Cartilage defect was created on the dorsal edge of the third carpal bone of one randomly selected intercarpal joint of each horse. The lesion was generated using a 5.0 mm × 13 cm arthroscopic burr.^2^ After the procedure, the debris was left in the joint, inducing synovitis and articular inflammation. Synovial membrane and joint capsule (approximately 3 mm × 3 mm) were harvested with a scalpel from the dorsal region of the joints for a study to be reported elsewhere. The sham-operated contralateral joints (SO) served as controls and were similarly evaluated by arthroscopic examination, synovial membranes and joint capsules were harvested but the cartilage was left intact. The arthroscopic portals were closed, forelimbs were bandaged, and horses were allowed to recover from the anaesthesia and surgery. The horses were housed in stall boxes. The status of each horse was monitored three times daily, including comfort, lameness at walk, body temperature, heart rate and respiratory rate.

Five days post-surgery, the lameness examination was repeated, new SF samples from both intercarpal joints were harvested and one randomly selected intercarpal joint was injected IA with 3 mL HA (20 mg/mL). Nine days after the IA HA injection (i.e. 2 weeks after the surgical arthroscopic procedure) the third lameness evaluation was done, and the third SF sampling, and the second synovial soft tissue sampling of both intercarpal joints were performed under general anesthesia, after which horses were euthanized on the operating table.

### Laboratory analyses

Synovial fluid samples obtained on day 0, day 5 and day 14 were used to analyze for the concentration of IL-1ra, PDGF-BB, TGF-β_1_ and TNF-α. All markers were assayed using commercial ELISA development kits^3^, ^4^, ^5^, ^6^ from R&D Systems. Samples were analysed in triplicate. TGF-β_1_ (see footnote 3) and PDGF-BB (see footnote 4) were determined using human antibodies. IL-1ra (see footnote 5) and TNF-α (see footnote 6) were assayed with equine-specific antibodies. The standards provided for each ELISA kit were used in preparing each standard curve according to the manufacturers’ instructions. Readings were performed at 450 nm. The inter-, and intra-assay coefficient of variation was < 6% for each ELISA.

### Statistical methods

IL-1ra, PDGF-BB, TGF-β_1_, TNF-α, WBC count and TP concentration were analysed with analysis of covariance models (ANCOVA). The study design had 3 time-points and 2 different interventions (operation, treatment). The two effects were analysed separately due to small sample size. The change in biomarker and TP concentrations and WBC count from pre-operation to pre-treatment was examined in one analysis and the change from pre-treatment to end of follow-up in another analysis. In both analyses, the change in concentration was used as the response, operation type or treatment as the fixed effect and the corresponding baseline measurement as a covariate.

As there were still some doubts about the normality of distributions after transformation, the changes in biomarker and TP concentrations and WBC count were analysed also using the Wilcoxon signed rank test. The Wilcoxon test was conducted also for all 16 limbs (effect of operation regardless of type). A P-value < 0.05 was accepted as statistically significant for all tests.

## Results

### Clinical outcomes

Five days after arthroscopy, all the horses showed signs of lameness and increased score in the flexion test of the CD-limb and effusion of the affected joint. The mean lameness score of the CD-limbs was 2.5, the mean flexion test score was 2.9 and the mean effusion score was 2.1, respectively. Two horses showed bilateral lameness, i.e. had a mildly lame SO-limb. In the SO-group, the mean scores of the flexion test and effusion were 1 and 1.8, respectively. No pain was elicited in the operated joint in maximal flexion in either of the groups (CD, SO).

**Fig. 1:**
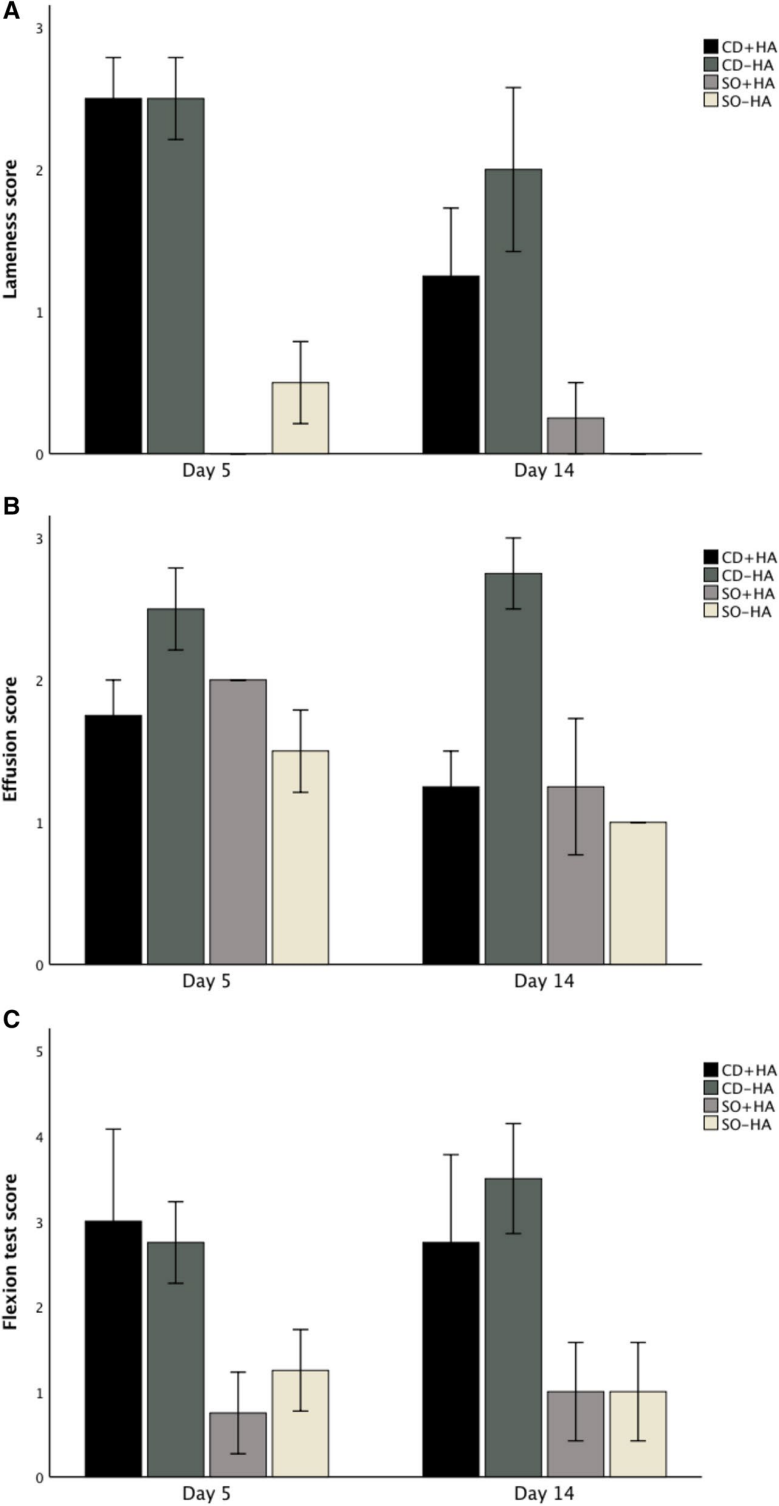
Mean (± SEM) clinical outcome measures of treatment groups post operation (day 5) and post treatment (day 14). *CD + HA* cartilage defect joints with hyaluronan injection, *CD − HA* cartilage defect joints without hyaluronan injection, *SO + HA* sham-operated joints with hyaluronan injection, *SO − HA* sham-operated joints without hyaluronan injection

Following trends can be seen in Fig. 1. After IA HA injection, the mean lameness score of the CD group with IA HA (CD + HA) decreased more compared to the group without HA-injection (CD − HA). Mild improvement in the flexion test score and more pronounced improvement in the effusion score were observed in the CD affected limbs of horses after IA HA (CD + HA) compared to the limbs without HA injections (CD − HA). Compared with CD groups, changes in the clinical scores and differences between the SO + HA and SO − HA groups were not consistent.

### Biomarker outcomes

In the ANCOVA models, no statistically significant differences in the Type III tests of fixed effects were documented: no effects were documented by the type of operation (CD vs. SO) or treatment (HA vs. no HA) to the concentrations measured in SF biomarkers. However, differences between the time points were revealed: the change from pre-operation to pre-treatment values were significant for IL-1ra (P = 0.0344 in the CD group and P = 0.0103 in the SO group), WBC count (P = 0.011 in the CD group and P = 0.002 in SO group) and TP (P = 0.0002 in the CD group and P = 0.002 in the SO group). HA had no significant effect on the biomarker concentrations within the groups.

In the Wilcoxon signed rank tests similar results were seen. The SF IL-1ra concentration was not significantly different for the operation types (CD vs. SO) but regarding all limbs the SF IL-1ra concentration increased significantly after arthroscopy (P = 0.0039). Also, the increase in SF TNF-α concentration was significant (P = 0.0386) regarding all limbs. Induction of CD and SO both caused a significant increase within both groups in WBC count (P < 0.001 in both groups) and TP concentration (P < 0.001 in both groups).

## Discussion

In the present study, the concentrations of the measured biomarkers in intact equine joints are mainly equivalent compared with the results of a previous report [20]. To the best of our knowledge, IL-1ra, PDGF-BB, TGF-β_1_ measured in the present study have been evaluated separately only in a few studies on SF of the equine joint [2, 21–23]. A purely catabolic cytokine TNF-α has been studied in greater detail [5, 20, 21, 23–28]. Most of the biomarkers have been documented in vitro in equine cartilage as well as in the synovial membrane after a challenge, usually lipopolysaccharide (LPS) [20, 29–31].

IL-1ra has been shown to increase after an acute intra-articular fracture in humans. However, differences in SF inflammatory cytokine concentrations between high and low-energy injuries have not been detected [32]. Although not directly comparable with intra-articular fractures, in the present study no differences were detected between CD joints and SO joints. However, the arthroscopy itself caused trauma to the synovial soft tissues and may have caused the increase of IL-1ra concentration.

Blocking the IL-1β receptor by IL-1ra has potentially a wide positive effect on inhibiting deleterious events in the joint. The binding of IL-1β to a receptor results in activation of several transcription factors and expression of hundreds of genes leading to the synthesis of other cytokines, chemokines, adhesion molecules, inflammatory mediators and enzymes [33]. Consequently, IL-1β is has a significant effect on the metabolism of cells and the extracellular matrix [34]. A decrease in SF IL-1ra has been documented in chronic stages of human OA [35]. Delayed increase of SF IL-1ra (at day 35) following the IA administration of autologous conditioned serum in experimentally induced equine OA has been demonstrated, suggesting endogenous production of IL-1ra [2]. In the present study, IA HA had no apparent effect on SF IL-1ra concentration in the short-term. However, long-term effects of IA HA on SF IL-1ra warrants further research.

PDGF is secreted in the early inflammatory phase primarily by platelets, but also by macrophages, endothelial cells and fibroblasts [36]. It is one of the earliest and the most sensible growth factors expressed after tissue injury [37]. PDGF induces the synthesis of other growth factors [38], proliferation and differentiation of fibroblasts, deposition of collagen and angiogenesis [39, 40]. Therefore, it is an essential promoter of the healing process. In the present study, after sampling at the baseline on day 0, the next sampling was performed on day 5, in a time point where PDGF-BB is supposed to play a major role in the vascular formation and proliferation of fibroblasts in ongoing repair [41]. PDGF-BB concentration increased in SF after induction of CD, although a significant difference was not detected. The synthesis of PDGF-BB may have, however, occurred earlier in the course of injury and inflammation. In contrast, PDGF-BB has not been detected at all in the SF of osteoarthritic human knee joint [42]. In another study comparing OA joints to healthy controls, significant differences in the SF PDGF-BB concentrations were not detected [43]. These results are also suggestive for an early increase of SF PDGF-BB concentration and its role in the initial phase of the pathogenesis of OA. SF PDGF concentration has been documented to increase after IA platelet rich plasma-treatment [23]. In the present study, no changes in the PDGF-BB concentrations were documented 9 days after IA HA treatment.

**Fig. 2:**
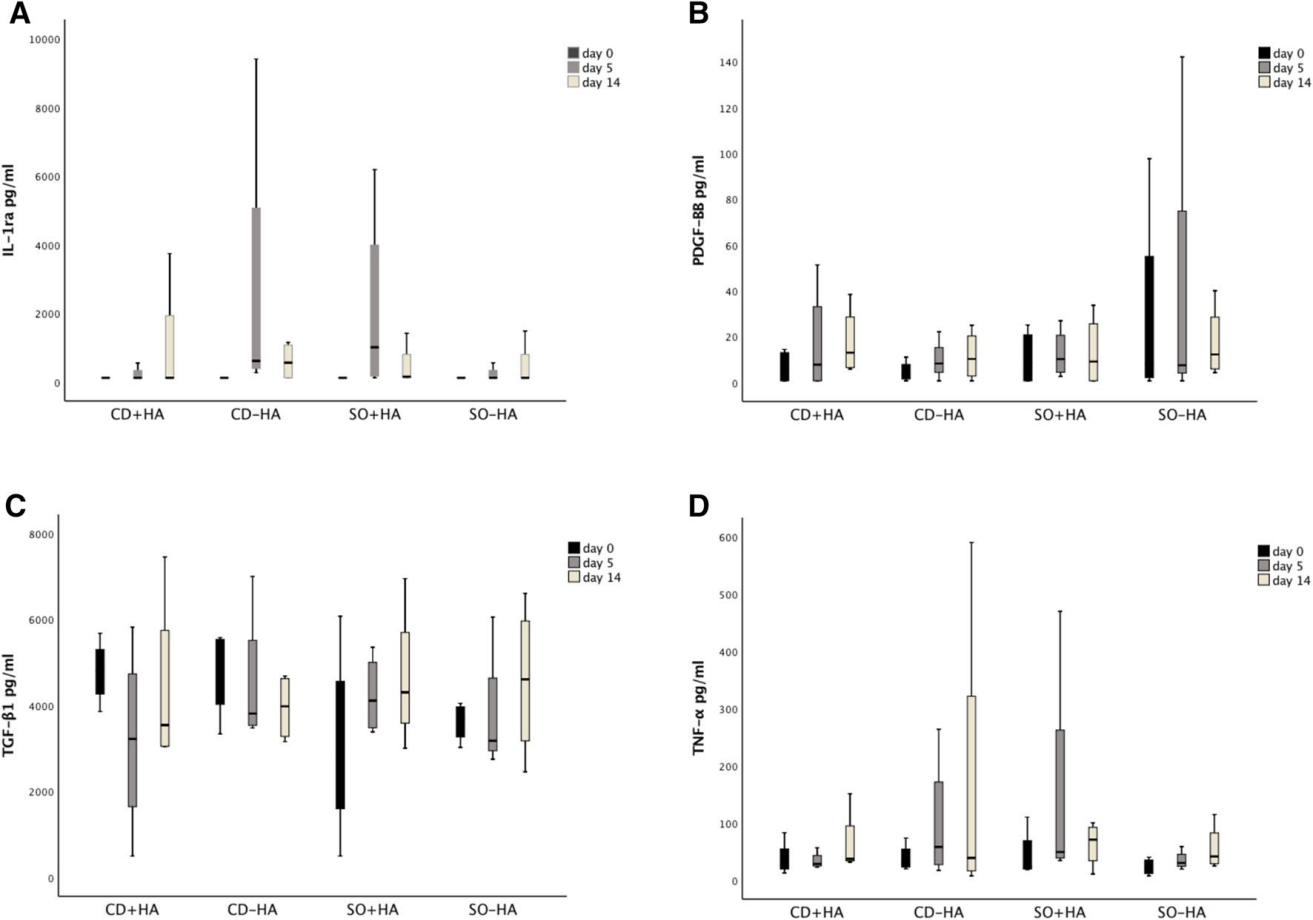
SF biomarker concentrations (median, quartiles and minimum and maximum value) of treatment groups on different sampling days. *CD + HA* cartilage defect joints with hyaluronan injection, *CD − HA* cartilage defect joints without hyaluronan injection, *SO + HA* sham-operated joints with hyaluronan injection, *SO − HA* sham-operated joints without hyaluronan injection

SF TGF-β_1_ has been studied in vivo in normal joints and joints with osteochondrosis in foals [22] and after LPS challenge in adult horses [21]. In the present study, neither the creation of CD or IA HA injection caused an increase in the SF TGF-β_1_ concentration. In contrast, TGF-β_1_ concentration decreased following the creation of CD, although no statistically significant difference between CD and SO joints was found (Fig. 2). This is different compared with the previous study on equine joints that reported increase of SF TGF-β_1_ concentration following a challenge with LPS [21]. This may be explained by the more intense inflammation induced by LPS, compared with inflammation induced by CD.

Ríos et al. [30] has shown an increase in TGF-β_1_ concentration after LPS challenge in cartilage inflammation created in vitro. The effect was suggested to result from a possible anti-inflammatory mechanism or by direct damage of LPS to the cartilage. A similar effect has been seen after LPS challenge of synovial membrane explants in vitro [31]. Our result is more consistent with the study of human OA where SF TGF-β concentration was measured both in healthy subjects and in patients with OA; in the latter, the concentration of SF TGF-β was low or even undetectable [44]. Cell signaling pathways may be intercepted by inflammatory cytokines, which may be a possible reason for the reduced amount of TGF-β in the course of OA [45].

TGF-β_1_ has an anabolic effect on cartilage; it has an ability to induce chondrogenic differentiation of mesenchymal stem cells [46, 47] with rapid biosynthesis of glycosaminoglycan and deposition of an extracellular matrix [47]. On the other hand, enhanced expression of TGF-β_1_ has been associated with developing osteophytes [48] and hyperplasia of the synovium [49]. It has been suggested that only a narrow range of bioactive TGF-β concentrations are beneficial to cartilage health and any concentrations below or above this range may cause aberrant alterations in TGF-β pathways, resulting in abnormal cartilage function [50]. As TGF-β is stored in the platelets, SF TGF-β concentration increases after IA administration of platelet rich plasma, in response to platelet activation [23]. HA has been suggested to play an important role in the mechanical activation of latent TGF-β in the joint [51]. However, in the present study, no changes in the SF TGF-β_1_ concentrations were documented after IA HA treatment, possibly implying that optimal SF TGF-β_1_ concentrations were already present in the injected joints.

The results of studies on the synovial fluid TNF-α are somewhat contradictory. However, it seems to be quite a sensitive but not very specific marker of IA insults. The results of this study are also suggestive of that. TNF-α concentration increased in SF after induction of CD although significant differences were not detected between the groups in the small population of this experimental study. An increased concentration of SF TNF-α has been documented in horses in naturally occurring OA in carpi [27], and experimentally in amphotericin B- [5] and LPS-induced articular inflammation [21, 26]. In horses, even exercise alone leads to a significant increase in TNF-α levels for a short period [25, 28]. However, an increase in SF TNF-α concentration as a result from serial arthrocentesis could not be demonstrated [25]. In contrast to this, gas and liquid capsular distension during arthroscopy provoked an inflammatory response with increased concentration of SF TNF-α [52]. In a clinical equine study, TNF-α was found not to be a useful biomarker for different types of joint lesions [53]. Similarly, in humans, increased concentrations were not associated with any particular type of articular disease, such as rheumatoid arthritis, although in OA patients, detectable concentrations of TNF-α were related with a long duration of the disease [24]. In the present study, SF TNF-α concentrations were not affected by IA HA. Probably with more horses, a bigger sample size and earlier and more frequent sampling significant results may have been demonstrated in this study.

In conclusion, after a mild increase in biomarker concentrations resulting from arthroscopy and induction of synovitis and CD, HA failed to produce any further effect on biomarkers. Minor improvement of clinical signs of IA inflammation was evident in the CD + HA group, when compared with scores of clinical signs of CD − HA group. However, in SO + HA and SO − HA groups changes after IA HA/no injection were not that consistent. HA is reported to have an anti-inflammatory effect [16] but IA HA injections have also been documented to induce a transitional IA inflammatory reaction, either a flare with pronounced clinical signs of inflammation and pain [54] or an increase of WBC count in the SF [11]. To our knowledge, only a few studies have explored biomarkers in equine SF after IA HA [3, 11]. As HA is very frequently used in the IA treatment in horses, its mechanism of action warrants further research.

This study has its limitations. Firstly, the contralateral limb of the horse served as a SO control. Concentrations of cartilage matrix products are elevated also in the contralateral knee in patients with anterior cruciate ligament rupture, possibly as a consequence of an altered loading [55]. Cytokines and degraded matrix products released from an operated joint may be transported to the contralateral joint by the circulation and initiate an inflammatory process. Therefore, the concentration of markers in the control joints may have resulted partly by the transport from the CD operated joint. Measurement of serum concentrations of studied markers, as well as additional pro-inflammatory markers and markers of cartilage matrix metabolism, would have provided additional information to test this hypothesis. However, this was outside the scope of this study. Moreover, the arthroscopy itself, as well as harvesting the synovial tissue samples, causes trauma to the synovial soft tissues. These may have further affected the concentrations of the measured markers in the SO joints. However, the effect was thought to be transient. To minimize the effect of arthroscopy and sampling of synovial soft tissue, sampling of SF and IA HA injection were performed only after 5 days.

The number of horses in the experimental studies on equine joints has generally ranged from 6 to 13 [1, 3, 5, 21, 26, 52]. Eight horses (16 joints) in the present study may have been too low to detect differences in concentrations of SF biomarkers. Although horses with uniform breed and sport discipline were selected, and all horses were free of lameness and joint disease of the intercarpal joint as verified by arthroscopy, differences in exercise or training status and age may have caused some variation in the SF biomarker concentrations. Exercise may lead to increased biomarker concentrations in horses [7, 28], which can be further enhanced in joints with compromised health [7, 25]. Moreover, also age has been reported to influence SF biomarker concentrations or gene expression so that they generally decrease with age [8, 29, 56].

The time from injury to collection of SF samples is an important issue as changes in biomarker concentrations can occur quickly, even within hours. On the other hand, IA injection itself, especially repeatedly, causes inflammatory reaction in the joint [57] and can cause increases in biomarker concentrations [56]. To minimize this effect, it has been recommended there should be a period of even 14 days after the previous arthrocentesis before subsequent SF collection [28]. However, despite of these potentially interfering factors, repeated sampling and early time points have been generally used in equine SF biomarker studies. In studies using LPS induction for IA inflammation, first sampling point 8 h [19] or 1 h [21, 26] post injection were chosen which is reasonable considering very acute and strong inflammation caused by LPS. On the other hand, in some experimental studies, using surgical model [3, 4] comparable with model of the present study or amphotericin B [5] for induction of IA inflammation in horses, weekly samplings were chosen for exploring SF IL-1ra and TNF-α concentrations (among other biomarkers), respectively. Finding an optimal time point for SF aspiration is challenging, especially when several biomarkers are studied. In addition, avoiding the effect of repeated aspiration and sham-operation on biomarker concentrations complicates the issue. Although the effect of IA injection on biomarkers measured in this study is not known, except for TNF-α, 5 days after arthroscopy and 9 days after IA HA injection were chosen to balance between the intervention time points.

## Conclusions

This study demonstrates that arthroscopy and both the creation of CD and SO, lead to an increase in SF IL-1ra and TNF-α concentrations but changes in concentrations of anabolic growth factors TGF-β_1_ and PDGF-BB in SF were not documented 5 days after the arthroscopy. Intra-articular HA was well tolerated. However, changes in concentrations of IL-1ra, PDGF-BB, TGF-β, and TNF-α were not seen after HA injection. The treatment effect of IA HA on SF pro-inflammatory, anti-inflammatory and anabolic biomarkers warrants further research.

## Data Availability

The datasets analysed during the current study are available from the corresponding author on reasonable request.

## Abbreviations

CD: cartilage defect
EDTA: etylenediaminetetraacetic acid
HA: hyaluronan
IA: intra-articular
IL-1ra: interleukin 1 receptor antagonist
LPS: lipopolysaccharide
NASHA: non-animal stabilized hyaluronic acid
OA: osteoarthritis
PDGF-BB: platelet-derived growth factor
PGE_2_: prostaglandin E_2_
SF: synovial fluid
SO: sham-operation
TGF-β_1_: transforming growth factor beta 1
TNF-α: tumour necrosis factor alpha
WBC: white blood cell

## Footnotes

1 Durolane, Bioventus LLC, Durham, NC, USA.

2 Dyonics Arthroscopic Surgery Blade, Smith & Nephew, Andover, MA, USA.

3 Human TGF-β1 DuoSet, DY240E, R&D Systems, Minneapolis, MN, USA.

4 Human PDGF-BB DuoSet, DY220, R&D Systems, Minneapolis, MN, USA.

5 Equine IL-1ra/IL1F3 DuoSet, DY1814, R&D Systems, Minneapolis, MN, USA.

6 Equine TNF-alphaDuoSet, DY1814, R&D Systems, Minneapolis, MN, USA.
